# The effects of protein supplementation and pasture maintenance on the growth, parasite burden, and economic return of pasture-raised lambs

**DOI:** 10.1093/tas/txab113

**Published:** 2021-06-29

**Authors:** Braden J Campbell, Antoinette E Marsh, Elizabeth M Parker, Jefferson S McCutcheon, Francis L Fluharty, Anthony J Parker

**Affiliations:** 1 Department of Animal Sciences, The Ohio State University, Wooster, OH 44691, USA; 2 College of Veterinary Medicine, The Ohio State University, Columbus, OH 43210, USA; 3 OSU Extension – Southeast Region, The Ohio State University, Caldwell, OH 43724, USA; 4 Department of Animal & Dairy Science, University of Georgia, Athens, GA 30602, USA

**Keywords:** gastrointestinal nematodes, grass-fed, grazing, sheep, haemonchus

## Abstract

The objective of this experiment was to evaluate the impact of protein supplementation and pasture contamination with gastrointestinal nematodes on the mitigation of parasitic infection in grazing lambs. We hypothesized that there would be no difference between protein supplementation and newly sown pasture in evaluating lamb growth and health parameters associated with parasitism. Furthermore, we questioned if there would be an interaction between protein supplementation and pasture type. A total of 192, 60-d-old lambs (28.3 ± 5.1 kg) were randomly assigned to one of four treatments: 1) new pasture without supplementation (NN); 2) new pasture with supplementation (NS); 3) established pasture without supplementation (EN); and 4) established pasture with supplementation (ES) and grazed for 112 d. Lambs were supplemented at a rate of 1% body weight/d. Supplemented lambs had greater body weight (BW) and average daily gain (ADG) when compared with non-supplemented lambs (*P* < 0.04). Additionally, lambs on newly sown pasture demonstrated greater BW and ADG when compared with lambs grazing on established pasture (*P* < 0.05). For lamb health, lambs in the EN treatment group had the greatest FAMACHA eye scores and lowest packed cell volume (PCV) over the course of the 112-d grazing period (*P* < 0.05). Moreover, NS and ES treatment lambs demonstrated similar FAMACHA eye scores when compared with NN treatment lambs; however, NN treatment lambs showed lower PCV when compared with NS and ES treatment lambs (*P* < 0.05). In evaluating fecal egg counts (FEC), lambs on new pasture or given supplement demonstrated lesser FEC when compared with those lambs on established pasture or not given supplement (*P* < 0.05). Sixty-four lambs were harvested to evaluate total abomasum nematode counts which demonstrated that *Haemonchus contortus* represented approximately 80% of total nematodes. Furthermore, based upon gross margin analysis, lambs given a protein rich supplement on pasture had a 9.3 kg increase in lamb BW whereas newly sown pasture had a 1.3 kg increase in lamb BW. A protein rich supplement given to lambs grazing pastures contaminated primarily with *H. contortus* or placing lambs on newly sown pasture increases lamb BW and improves parasite resiliency. Selection of parasite management strategies may be influenced by cost of production and market opportunities.

## INTRODUCTION

Gastrointestinal parasitic infection coupled with the continual development of anthelmintic resistance remains as a global concern for those that rear small ruminants using pasture-based systems (Waller 1997; Waller and Chandrawathani, 2005; Taylor, 2012; Torres-Acosta et al., 2012). Regardless of location, producer surveys disclose that the greatest contributor of anthelmintic resistance is the continued or improper use of anthelmintics (Calvete et al., 2012; Falzon et al., 2013). Previous effectiveness of antiparasitic products has led to the predominant use of anthelmintics on-farm to control for reduced animal growth and health losses as a result of parasitic infestation (Kaplan and Vidyashankar, 2012). However, alternative management practices to mitigate the negative effects associated with parasitic nematodes must be considered to decrease the industry’s reliance upon antiparasitic drugs. Management considerations that may aid in reducing the need for anthelmintics are those that focus on systems management, including but not limited to protein supplementation and pasture renovation.

In intensive grazing management systems, grazing is considered a high-risk practice when factors of forage quality and pasture parasite burden are unknown (Poli et al., 2020). Providing lambs grazing on pastures of poor quality with supplemental plant-derived protein primarily using soybean meal has shown to increase lamb body weight (BW) gain (Wallace et al., 1995; Wallace et al., 1996; Kahn et al., 2000; Felix et al., 2012). Improvements in lamb growth is credited to supplemental protein meeting or exceeding the increased protein demands required by young growing lambs (NRC, 2007). Furthermore, protein supplementation from plant-derived sources has also demonstrated improvements in lamb parasite resilience as supported by an increase in lamb packed cell volume (PCV) and decreases in lamb fecal egg count (FEC) and total nematode counts when compared with non-supplemented counterparts (Wallace et al., 1996; Houdijk et al., 2012; Crawford et al., 2020).

Producers may consider reducing nematode exposure and subsequent larvae intake by allowing pastures to remain fallow from grazing events or by establishing new pastures to break the reproductive life cycle of parasitic nematodes. Ideally, placing non-parasitized sheep on a pasture with minimum parasitic nematodes would greatly reduce the exposure of parasitic larvae to grazing lambs (Michel, 1985). However, factors beyond management control (i.e., precipitation, air temperature, relative humidity, and wildlife interactions) makes it impossible to develop and maintain a pasture free of parasitic nematodes (Waller, 2006). To produce a pasture with a low density of parasitic nematodes, supported by low lamb FEC’s over a 4-wk intensive grazing period, can be achieved by removing sheep from pasture for 2 yr (Kidane et al., 2010). Furthermore, pasture renovation techniques using soil cultivation have also shown to decrease the density of viable parasitic larvae found on pasture (Moss et al., 2011).

Developing and implementing alternative parasite management practices are crucial for the sustainability of grass-fed lamb operations. Providing supplemental protein to grazing lambs as well as converting cropland to newly sown pasture may serve as additional strategies to reduce the need for anthelmintic use. Thus, the objective of these experiments was to evaluate the effect of protein supplementation and pasture contamination with gastrointestinal nematodes on the growth and health parameters of grazing lambs. We hypothesized that there would be no difference between protein supplementation and newly sown pasture in evaluating lamb growth and health parameters associated with parasitism. Furthermore, we questioned if there would be an interaction between protein supplementation and pasture type.

## MATERIALS AND METHODS

The Ohio State University Animal Care and Use Committee approved the protocol for this experiment (#2017A00000029). Animals were cared for in accordance with the United States Animal Welfare Act and the Guide for the Care and Use of Agricultural Animals in Agricultural Research and Teaching (FASS, 2010).

## ANIMALS, HOUSING, AND HUSBANDRY

### Animals

A total of 192 spring born Dorset × Hampshire, Dorset × Suffolk, Hampshire × Dorset, and Suffolk × Dorset, crossbred lambs from the Ohio Agricultural Research and Development Center (OARDC) Sheep Unit Wooster, Ohio were evaluated over the course of 2 yr (2018, 2019). Each year, grazing events were initiated in June and concluded in October with a total grazing period of 112 d. Lambs (ewes and wethers) were weaned at 60 d of age, with an initial average starting BW of 28.3 ± 5.1 kg. Lambs were born, reared, and housed indoors for the first 60 d of life to reduce exposure to parasitic infection prior to the initiation of the grazing periods. As a result, prior to each experimental grazing period, lambs were not exposed to parasitic infection nor treated with an anthelmintic product. Lambs were stratified by BW and sex, then randomly assigned to one of four treatment groups. Treatments were allocated in a 2 × 2 factorial design where lambs were assigned to the following treatments: 1) new pasture without supplementation (NN); 2) new pasture with supplementation (NS); 3) established pasture without supplementation (EN); and 4) established pasture with supplementation (ES). Treatment groups were replicated four times each year, with six lambs per replicate (24 lambs/treatment group/yr).

### Pasture Management

Lambs assigned to the NN and NS treatment groups were placed on a new pasture, described as newly sown pasture converted from cropland that had never been fertilized with livestock manure nor grazed by sheep or other ruminant livestock. Lambs assigned to the EN and ES treatment groups were placed on established pasture, described as permeant sheep pasture that is rotationally grazed year-round by the existing OARDC ewe flock. Forages in both pastures were primarily dominant (90%) in tall fescue (*Schedonorus arundinaceus* (Schreb.)). Paddocks were rotationally grazed every three days as described by Campbell et al. (2017). Lambs assigned to the NS and ES treatment groups were supplemented with a plant-derived protein rich supplement (Table 1). Supplement treated groups were given supplement in a portable feed trough (PortaTrough 5, Premier 1 Supplies, Washington, IA, USA) at a feeding rate of 1% live BW/d, with supplementation rates adjusted every 14 d to reflect the most recent average group BW.

**Table 1.** Ingredient (%) and nutrient composition (%) of protein supplement given to supplement treated lambs at an intake of 1% live body weight per day

Item	Supplement	
*Ingredient* *		
Corn gluten meal	30.00	
Distillers dried grains with solubles	37.62	
Soybean meal	30.34	
Fat	2.00	
Amaferm (64 g/lb. in conc.)	0.04	
Year	2018	2019
*Calculated nutrient composition* ^*1*^		
Crude protein	47.31	49.01
Acid Detergent Fiber	10.99	8.82
Neutral Detergent Fiber	15.36	17.52
Calcium	0.13	0.15
Phosphorous	0.59	0.61

^*^Parameters calculated and reported as a percentage on a dry matter basis.

Pastures (new and established) were approximately 2.5 ha in size. Each 2.5-ha pasture was divided into eight equal size paddocks each year. Furthermore, each paddock was further divided into sub-paddocks, which were calculated based upon equal stocking density or equal live animal weight per hectare. Sub-paddock sizing was subject to change based upon live lamb BW and or forage dry matter production over the duration of the grazing period. Portable electric fencing (VersaNet Plus, Premier1Supplies, Washington, IA, USA) was used to construct each grazing paddock and sub-paddocks within the permanent 2.5-ha pasture. Pastures in the EN and ES treatment groups were given 35 d of rest and re-growth prior to the next grazing session to mimic a rotational grazing system. Pastures in the NN and NS treatment groups were grazed for a 3-d period with lambs never returning to the same grazing area during the entirety of the grazing season. Mechanical clipping was practiced to ensured that the new pasture was of similar dry matter availability and forage residual height when compared with the established pasture. Furthermore, ad libitum access to water and mineral (Purina Wind & Rain Sheep Mineral, Purina Animal Nutrition LLC, Shoreview, MN, USA) were provided in each paddock and monitored daily.

### Environmental Conditions

**Figure 1.**
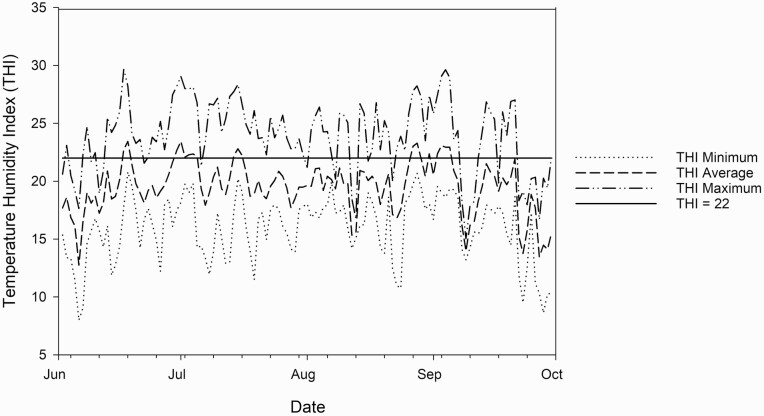
Daily minimum, averge, and maximum temperature humidity index (THI) summary for lambs grazing from June to October in 2018 at the Ohio Agriculture Research and Development Center in Wooster, Ohio, USA. The THI 22 reference line illustrates the THI value considered to be heat stress for sheep (Lees et al. 2019).

Daily low, average, and high ambient air temperature was recorded using The Ohio State University’s College of Food, Agriculture, and Environmental Sciences weather monitoring system for the OARDC Wooster location. These data were used to develop a Temperature Humidity Index (THI) for the 2018 grazing period (Figure 1). However, there was a malfunction in the weather station’s relative humidity sensor for the OARDC Wooster location that resulted in no values for relative humidity recorded in 2019 and therefore a THI was not calculated for this grazing period.

### Forage Measurements

Forage dry matter allowance was measured every 2 wk on d 1, 15, 29, 43, 57, 71, 85, 99, and 113 by clipping forage to ground level using hand shears within a 0.66 m^2^ quadrat. A subsample of quadrat clippings was dried at 100^o^ C for 48 h. to calculate forage dry matter availability per hectare.

To assess forage quality, 24 random grab samples were collected on the same days as noted above. Grab samples were collected in a manner that mimicked lamb grazing behavior and were derived from paddocks in which lambs would be entering on their next scheduled move. Forage quality samples were analyzed for moisture, crude protein (CP), acid detergent fiber (ADF), neutral detergent fiber (NDF), minerals (Ca, P, K, Mg, and S), relative feed value (RFV), and total digestible nutrients (TDN; Rock River Laboratory, Inc., Wooster, OH, USA).

## LAMB GROWTH AND HEALTH

### Lamb Growth

Lamb BW was collected on d 0, 14, 28, 42, 56, 70, 84, 98, and 112 of the grazing period each year. Body weights were collected using a digital and pneumatic handling system (DR3 Te Pari Racewell Auto Drafting system, Te Pari Products Ltd, Oamaru, New Zealand). Average Daily Gain (ADG) was calculated by taking the difference in lamb BW from the consecutive sampling period and dividing by the number of days between each period.

### Lamb Health

Lamb FAMACHA eye scores, PCV, and FEC samples were collected on the same days as previously outlined in the lamb growth section. These data were collected using the same sampling techniques as described by Campbell et al. (2017).

Anthelmintic treatment for grazing lambs was based upon university livestock facility standard operation procedures. Sheep that displayed PCV values of ≤21% were treated with an anthelmintic recommended by the attending university veterinarian. In 2018, lambs in need of anthelmintic treatment received moxidectin (Cydectin Oral Sheep Drench, Boehringer Ingelheim, Ingelheim am Rhein, Germany) at a rate of 0.2 mg/kg BW. Upon evaluation of anthelmintic efficacy on-farm, lambs in need of a secondary treatment because of decreased PCV values received levamisole hydrochloride (Prohibit, AgriLabs, Agri Laboratories, Ltd., St. Joseph, MO, USA) at a rate of 8 mg/kg BW. In 2019, lambs in need of one or more anthelmintic treatments received levamisole hydrochloride at 8 mg/kg BW.

## GASTROINTESTINAL PARASITE EVALUATION

### Gastrointestinal Parasite Quantification and Speciation

Two lambs from each replicate (8 lambs/treatment group/yr) were randomly selected at the conclusion of each grazing period, removed from pasture, and transported to The Ohio State University abattoir for harvest. Upon stunning and exsanguination, lambs were eviscerated to remove the abomasum and large intestine. Lamb abomasa were incised along the greater curvature with all contents removed and placed into a labeled container. Sampling aliquots and abomasa soaking procedures for nematode quantification are further described by Wood et al. (1995). For each lamb, a 2 cm wide strip of abomasal wall was dissected approximately 4 cm above the pyloric sphincter and immediately placed in 10% formalin solution. The large intestine was collected from each harvested lamb to obtain a final fecal sample. Peanut lectin staining (Palmer and McCombe, 1996) was used to fluoresce fecal eggs with eggs staining positive speciated and recorded as *Haemonchus contortus* (Jurasek et al., 2010).

### Histology

Abomasal tissue was fixed in 10% formalin solution and was processed by routine histological techniques performed by the Comparative Pathology and Mouse Phenotyping Shared Resource laboratory at The Ohio State University College of Veterinary Medicine. Three sections of 6 µm tissue were stained with hematoxylin-eosin and toluidine blue for evaluation of inflammatory cell infiltration. Tissue histology samples were evaluated visually, with samples categorized as demonstrating no evidence of an inflammatory response or an overt response. Furthermore, the author evaluating the histological sections was blinded to treatment.

### Statistical Analysis

Data on lamb growth and health parameters were analyzed utilizing SAS software (SAS version 9.4, SAS Institute Inc., Cary, NC, USA). A generalized linear mixed model method (PROC MIXED) with a Kenward–Roger approximation for degrees of freedom was used to determine differences among treatment groups. The model included the fixed effects of pasture type (new or established), supplementation (with or without), day, and their interactions. The random statement included year, sex × weight, and group nested within pasture. Repeated measures of lamb body weight, average daily gain, packed cell volume, fecal egg counts, and FAMACHA eye score were based upon day of collection. LSMEANS and PDIFF were used to determine treatment least square mean estimates and pooled standard errors of the mean. Data are reported as means ± SEM with significance being determined using a *P*-value <0.05. Over the course of the 2-yr grazing period, a total of eight lambs, four from each respective year, were removed from the experiment due to complications associated with poor growth rates coupled with parasitic infection and thus excluded from the final analysis.

## RESULTS

### Forage Quality Measurements

Forage quality parameters presented by pasture (new and established) and year (2018 and 2019) are presented in Table 2. Data have not been statistically analyzed and therefore are used for descriptive purposes only. All nutrients are reported on a dry matter (DM) basis.

**Table 2.** Pasture dry matter (DM), crude protein (CP, %), acid detergent fiber (ADF, %), neutral detergent fiber (NDF, %), and total digestible nutrients (TDN, %) values for new and established pastures in 2018 and 2019, respectively

Item	New	Established	New	Established
Year	2018		2019	
DM				
d 1	22.34	20.09	24.35	25.38
d 15	19.74	19.54	27.45	25.72
d 29	29.61	24.24	25.69	25.99
d 43	31.39	26.57	29.12	26.27
d 57	21.14	24.08	27.56	24.92
d 71	28.07	24.91	29.08	24.22
d 85	27.01	24.76	27.96	25.33
d 99	27.98	26.19	25.01	24.81
d 113	25.28	15.1	26.76	24.36
CP^*^				
d 1	10.61	14.49	14.03	13.45
d 15	12.26	13.08	11.07	13.90
d 29	8.73	13.59	10.53	13.74
d 43	8.17	11.33	11.11	13.74
d 57	10.13	13.52	10.22	13.57
d 71	9.39	13.64	11.06	17.04
d 85	10.02	15.76	15.33	18.25
d 99	10.87	14.68	16.33	18.35
d 113	13.66	19.85	13.97	18.71
ADF^1^				
d 1	36.35	31.65	27.35	29.22
d 15	34.50	33.29	28.47	28.33
d 29	35.56	32.23	32.51	28.83
d 43	37.81	34.47	32.01	29.07
d 57	35.72	32.93	33.02	29.67
d 71	33.46	31.29	37.20	29.51
d 85	34.23	33.54	31.55	30.97
d 99	36.13	33.79	29.23	30.35
d 113	30.35	32.49	29.84	30.89
NDF^1^				
d 1	60.33	53.43	45.43	48.60
d 15	58.07	57.02	49.53	49.54
d 29	58.95	57.71	50.26	50.21
d 43	58.34	55.93	50.91	48.88
d 57	58.43	57.10	55.62	52.38
d 71	52.55	52.65	59.69	51.49
d 85	57.09	53.76	54.31	51.83
d 99	57.15	55.35	54.64	50.25
d 113	55.19	53.14	52.60	54.43
TDN^*^				
d 1	65.30	52.93	77.15	72.54
d 15	55.74	52.59	71.08	74.82
d 29	61.02	57.36	56.98	68.37
d 43	63.41	54.21	54.27	64.78
d 57	61.85	51.44	51.47	65.50
d 71	59.52	70.06	64.51	70.41
d 85	60.93	70.48	67.55	78.77
d 99	54.12	58.50	76.41	73.83
d 113	65.16	73.53	71.68	73.54

^*^Pasture CP, ADF, NDF, and TDN calculated on a dry matter basis.

### Lamb Growth

Data representing lamb growth is presented in Table 3. There was no significant pasture × supplement × day interaction detected for lamb BW and ADG (*P* > 0.05). However, there was a pasture × day effect for lamb BW and ADG. For lamb BW, on d 14, 28, and 56, lambs grazing established pasture demonstrated greater BW when compared with lambs grazing new pasture (*P* ˂ 0.002). On d 98 and 112, however, lambs grazing established pasture exhibited lesser BW when compared with lambs grazing new pasture (*P* ˂ 0.05). For lamb ADG, on days 14 and 56, lambs grazing established pasture had a greater ADG when compared with lambs grazing new pasture (*P* ˂ 0.0009). Conversely, on d 28, 42, 70, and 98, lambs grazing new pasture showed a greater ADG when compared with lambs grazing established pasture (*P* ˂ 0.0001).

**Table 3.** Mean ± SEM body weight (kg) and average daily gain (ADG^2^, g/day) for lambs grazing new or established pastures and either supplemented or non-supplemented

Item	Pasture			Supplementation		
	New	Established	SEM^*^	Supplemented	Non-supplemented	SEM^*^
Body weight			2.53			2.54
d 0	28.4	28.3		28.3	28.4	
d 14	24.8^a^	28.3^b^		27.4^a^	25.8^b^	
d 28	28.8^a^	30.5^b^		31.1^a^	28.2^b^	
d 42	29.6	30.2		32.1^a^	27.7^b^	
d 56	31.3^a^	32.8^b^		34.9^a^	29.2^b^	
d 70	33.1	32.9		36.8^a^	29.2^b^	
d 84	33.6	33.8		37.8^a^	29.6^b^	
d 98	34.4^a^	33.4^b^		38.4^a^	29.5^b^	
d 112	36.8^a^	35.4^b^		40.7^a^	31.4^b^	
ADG^2^			19			17
d 14	−254^a^	−1^b^		−66^a^	−190^b^	
d 28	281^a^	157^b^		264^a^	174^b^	
d 42	59^a^	−25^b^		72^a^	−38^b^	
d 56	121^a^	190^b^		198^a^	113^b^	
d 70	130^a^	8^b^		137^a^	1^b^	
d 84	39	62		73^a^	28^b^	
d 98	57^a^	−27^b^		42^a^	−11^b^	
d 112	168	137		169	137	

^a^,

^b^ Means within a row with different superscripts differ (*P* < 0.05).

^*^ Pooled standard error of the mean.

Additionally, there was a supplementation × day effect for lamb BW and ADG. For lamb BW, on d 14, 28, 42, 56, 70, 84, 98, and 112, supplemented lambs demonstrated greater BW when compared with non-supplemented lambs (*P* ˂ 0.0008). For lamb ADG, on d 14, 28, 42, 56, 70, 84, and 98, supplemented lambs had a greater ADG when compared with non-supplemented lambs (*P* ˂ 0.04).

### Lamb Health

Data describing lamb health are provided in Tables 4 and 5. Data in Table 4 outline lamb FAMACHA eye score and PCV. There was a significant pasture × supplement × day interaction for lamb FAMACHA eye score and PCV. For lamb FAMACHA eye score, on d 14, lambs in the ES treatment group had lesser FAMACHA eye scores when compared with all other treatment groups (*P* < 0.02). On d 28, lambs in the NN and ES treatment groups had lesser FAMACHA eye scores when compared with the EN treatment group (*P* < 0.04), whereas lambs in the NS treatment group did not differ from all treatment groups (*P* > 0.05). Furthermore, on d 42, 56, 70, 84, and 112, lambs in the EN treatment group had greater FAMACHA eye scores when compared with all other treatment groups (*P* < 0.02). On d 98, lambs in the EN treatment group had the greatest FAMACHA eye scores when compared with all other treatment groups (*P* < 0.05). Moreover, on d 98, lambs in the NN treatment group had greater FAMACHA eye scores when compared with the NS treatment group (*P* < 0.05), whereas lambs in the ES treatment group did not differ from the NN or NS treatment groups.

**Table 4.** Mean ± SEM FAMACHA eye score and packed cell volume (%) for lambs treated with new pasture without supplementation (NN), new pasture with supplementation (NS), established pasture without supplementation (EN), or established pasture with supplementation (ES)

Item	NN	NS	EN	ES	SEM^*^
FAMACHA^†^					0.35
d 0	1.1	1.1	1.2	1.0	
d 14	1.3^a^	1.5^a^	1.3^a^	1.1^b^	
d 28	1.3^a^	1.6^a^^,^^b^	1.8^b^	1.4^a^	
d 42	1.2^a^	1.3^a^	2.3^b^	1.4^a^	
d 56	1.2^a^	1.2^a^	1.9^b^	1.5^a^	
d 70	1.4^a^	1.3^a^	2.3^b^	1.5^a^	
d 84	1.6^a^	1.4^a^	2.8^b^	1.7^a^	
d 98	1.6^a^	1.3^b^	3.1^c^	1.3^a^^,^^b^	
d 112	1.4^a^	1.2^a^	2.6^b^	1.3^a^	
Packed cell volume					0.93
d 0	35.0	35.1	35.3	35.9	
d 14	38.4^a^	37.0^b^	39.2^a^	37.8^a^^,^^b^	
d 28	38.3^a^	36.3^b^	33.8^c^	34.7^c^	
d 42	40.9^a^	40.0^a^	30.0^b^	34.7^c^	
d 56	33.5^a^	33.2^a^	25.8^b^	27.9^c^	
d 70	29.7^a^	30.8^a^	22.4^b^	26.4^c^	
d 84	28.0^a^	30.1^b^	20.1^c^	26.7^a^	
d 98	26.6^a^	30.2^b^	21.3^c^	29.7^b^	
d 112	26.4^a^	30.2^b^	23.0^c^	29.1^b^	

^a^,

^b^,

^c^ Means within a row with different superscripts differ (P < 0.05).

^*^ Pooled standard error of the mean.

^†^FAMACHA Eye Score color chart: ‘1’ = red, non-anemic mucous membrane; ‘2’ = red- pink, non-anemic mucous membrane; ‘3’ = pink, mildly anemic mucous membrane; ‘4’ = pink-white, anemic mucous membrane; ‘5’ = white, severely anemic mucous membrane.

**Table 5.** Mean ± SEM fecal egg count (eggs/g) for lambs grazing new or established pastures and either supplemented or non-supplemented

Item	Pasture			Supplementation		
	New	Established	SEM^*^	Supplemented	Non-supplemented	SEM^*^
Fecal egg count						
Transformed, log(*x* = 10)			0.27			0.24
d 0	2.3	2.3		2.3	2.3	
d 14	2.5	2.7		2.6	2.5	
d 28	2.7^a^	7.3^b^		4.7^a^	5.2^b^	
d 42	3.0^a^	7.4^b^		5.0^a^	5.5^b^	
d 56	2.6^a^	7.2^b^		5.1	4.7	
d 70	2.8^a^	7.5^b^		5.3	5.0	
d 84	2.8^a^	5.8^b^		4.1	4.4	
d 98	3.0^a^	4.9^b^		3.8	4.0	
d 112	2.7^a^	4.7^b^		3.5^a^	4.0^b^	
Back transformed			–			–
d 0	0	0		0	0	
d 14	3	4		4	3	
d 28	4	1482		104	178	
d 42	10	1672		134	223	
d 56	3	1383		153	105	
d 70	7	1713		189	136	
d 84	6	317		53	74	
d 98	10	121		36	47	
d 112	5	101		23	43	

^a^,

^b^ Means within a row with different superscripts differ (P < 0.05).

^*^ Pooled standard error of the mean.

For lamb PCV, on d 14, lambs in the NN and EN treatment groups had greater PCV values when compared with the NS treatment group (*P* < 0.05), however, lambs in the ES treatment group did not differ from all treatment groups (*P* > 0.05). On d 28, lambs in the NN treatment group demonstrated the greatest PCV values, with lambs in the EN and ES treatment groups having the lowest PCV values and lambs in the NS treatment group being intermediate (*P* < 0.04). Additionally, on d 42, 56, and 70, lambs in the NN and NS treatment groups had the greatest PCV values, whereas lambs in the ES treatment group had the lowest PCV values and lambs in the EN treatment group were intermediate (*P* < 0.02). Furthermore, on d 84, lambs in the NS treatment group had the greatest PCV values when compared with all other treatment groups, with NN and ES treatment lambs demonstrating greater PCV values when compared with EN treatment lambs (*P* < 0.005). Finally, on d 98 and 112, lambs in the NS and ES treatment groups had the greatest PCV values when compared with all other treatment groups, whereas lambs the NN treatment group had greater PCV values when compared with the EN treatment group (*P* < 0.001).

Data in Table 5 represent lamb FEC. There was no significant pasture × supplement × day interaction detected for lamb FEC (*P* = 0.0544). A pasture × day interaction, however, was demonstrated for lamb FEC in that on d 28, 42, 56, 70, 84, 98, and 112 as lambs grazing new pasture had lesser FEC when compared with lambs grazing established pasture (*P* < 0.0001). Additionally, there was a supplementation × day interaction for lamb FEC whereas on days 28, 42, and 112, supplemented treatment lambs exhibited lesser FEC when compared with non-supplemented lambs (*P* < 0.05).

Over the course of the 2-yr grazing experiment, a total of 32 and 34 lambs were treated for parasitic infection in 2018 and 2019, respectively. Over both years, zero lambs from the new pasture treatment group required anthelmintic treatment. Of those lambs treated in 2018, 10 lambs received two doses of anthelmintics. In 2019, of those lambs treated, seven lambs received two doses of anthelmintics whereas five lambs received three doses of anthelmintics.

### Nematode and Histological Data

**Figure 2.**
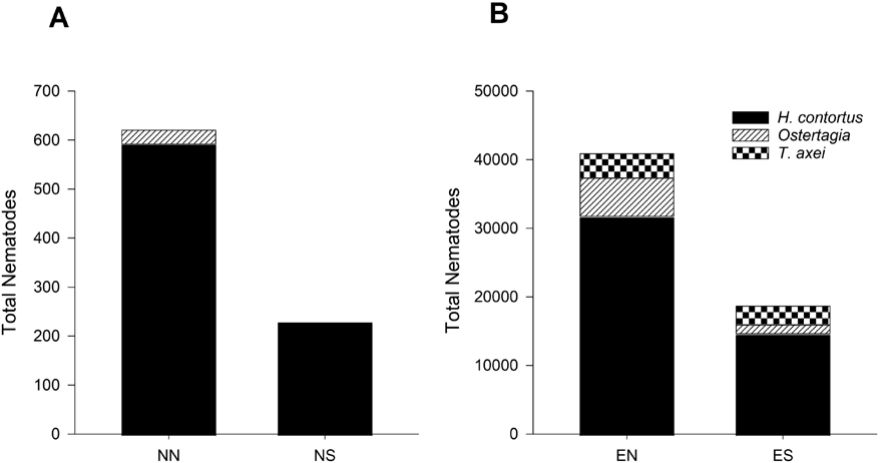
Total abomasal nematode counts for lambs treated with (A) new pasture without protein supplementation (NN) and new pasture with protein supplementation (NS) or (B) lambs treated with established pasture without protein supplementation (EN) and established pasture with protein supplementation (ES). Nematodes of interest included *Haemonchus contortus*, *Ostertagia*, and *Trichostrongylus axei*.

Total gastrointestinal nematode counts and proportions of nematode species are shown in Figure 2. Total gastrointestinal nematode counts represent all nematodes collected from the abomasum. Furthermore, based upon fecal egg fluorescence, 66% and 59% of fecal eggs evaluated immediately after lamb harvest in 2018 and 2019, respectively, were identified as *H. contortus*.

**Figure 3.**
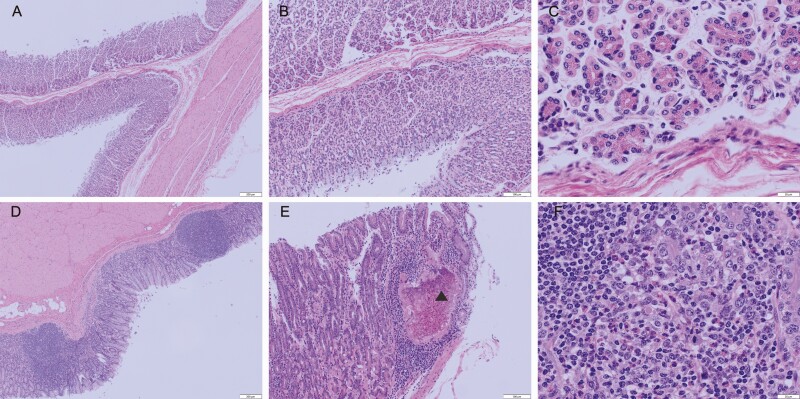
(A–C) Abomasum of lambs grazing new pastures lacks inflammatory infiltrates in mucosa, lamina propria, and submucosa. (D–F) Abomasum of lambs grazing established pastures with prominent granulomatous inflammation characterized by lymphocyte, macrophage, and eosinophil infiltration in mucosa, lamina propria, and submucosa. The arrowhead in (E) (▲) indicates an area of necrosis surrounded by granulomatous inflammation.

The abomasal tissue of sampled lambs that grazed on new pasture were histologically normal with no evidence of an inflammatory response (Figure 3A–C). The mucosa, lamina propria, and submucosa of abomasal tissue collected from lambs that grazed on established pasture were diffusely infiltrated with lymphocytes, plasma cells, macrophages, and eosinophils with focal areas of granulomatous inflammation and necrosis (Figure 3D–F). There was no difference in mast cell infiltration between lambs that grazed on new or established pasture. There was also no difference detected in the histological sections between supplemented and non-supplemented lambs. Pathology data were not statistically analyzed because of the stark contrast created by the pasture treatments but are reported to support other health data.

### Economic Analysis

Table 6 provides estimates of net value of lamb produced on pasture based upon treatment. Data provided in this table are used to descriptively support the use of each management strategy. Estimates and assumptions provided may vary based upon geographical location and market demand.

**Table 6.** Lamb net value calculations based upon mean lamb final body weight (BW, kg), estimated lamb sale value (USD, $), pasture establishment and/or maintenance costs (USD, $), and supplementation costs (USD, $) for lambs grazing new or established pastures and either supplemented or non-supplemented

Item	Pasture		Supplementation	
	New	Established	Supplemented	Non-supplemented
Starting BW^a^	28.4	28.3	28.3	28.4
Final BW^b^	36.8	35.4	40.7	31.4
BW gain^c^	8.4	7.1	12.4	3.0
Price/kg of BW^d^	3.61	3.61	3.61	3.61
Net value of BW gain^e^	30.32	25.63	44.76	10.83
Pasture Cost/lamb^f^	8.16	4.49	6.33	6.33
Supplement Cost/lamb^g^	8.47	8.47	16.94	0.00
Net Value/Lamb	13.69	12.67	21.49	4.50

^a^Lamb starting BW based upon day 0 from Table 3.

^b^Lamb final BW based upon day 112 from Table 3.

^c^Lamb BW gain over the course of the 112-day grazing period.

^d^Lamb price/kg = Final BW (kg) × Mean sale price / kg of BW. Sale price data gathered from Mt. Hope auction reports from October 3, 2018 (http://www.mthopeauction.com/sites/default/files/market_reports/LMR%20Market%20Report10-3–18.pdf) and October 2, 2019. (http://www.mthopeauction.com/sites/default/files/market_reports/LMR%20Market%20Report10-2–19.pdf). The mean of the reported range was taken as the price for 60#–80# lambs (range of prices = $3.08–$4.14/kg.). (Accessed: 18 February 2021).

^e^Net value of BW gain = BW × price/kg of BW.

^f^Pasture cost/lamb based upon pasture type. New pasture costs calculated using values for seed purchase, soil tillage, seed planting, fertilizer purchase, fertilizer application, and pasture maintenance using mechanical clipping. Established pasture costs included fertilizer purchase, fertilizer application, and pasture maintenance using mechanical clipping. Pasture costs were averaged an applied to the treatment of supplementation to account for both pasture treatments listed within supplementation treatments. Custom agricultural production estimates were calculated using 2020 Ohio Farm Custom Rates (https://farmoffice.osu.edu/sites/aglaw/files/site-library/farmBusiness/Ohio%20Farm%20Custom%20Rates%20Draft%202020%20Final%20Revised.pdf). Seeding rate estimates are provided by The Ohio State University Agronomy Guide (https://stepupsoy.osu.edu/sites/hcs-soy/files/472%20Ohio%20Agronomy%20Guide%2015%20Ed%20red_0.pdf). Seed costs estimates for tall fescue (Kentucky-31) were averaged using pricing from three USA retailers for a 50 lb. bag (Rural King, TSC, and Ace Hardware). Fertilizer estimates for Urea (46-0-0) were averaged using pricing from three USA retailers for a 50 lb. bag (Amazon, Wal-Mart, and Seed World). Pasture pricing assumption do not include land costs. (Accessed: 22 February 2021).

## DISCUSSION

We accept our hypothesis that there would be no difference between protein supplementation and pasture type for lamb growth and health parameters associated with parasitism. Our justification for the acceptance of our hypothesis is demonstrated by improvements in lamb growth and economic return based on grazing treatment as well as a decrease in lamb fecal egg output and total worm burden when compared with non-supplemented and established pasture treatments. Furthermore, we did report interactions between protein supplementation and pasture type when evaluating lamb FAMACHA eye score and PCV. However, these interactions should be interpreted with caution. Results from the current study demonstrate that *H. contortus* was the main nematode of concern as highlighted by fecal egg fluorescence and nematode speciation. These data confirm the appropriate use of the FAMACHA eye scoring system in the current study and also demonstrate the importance of identifying parasite species in mixed populations to determine which diagnostic tools are most appropriate to use when evaluating overall parasite burden. Moreover, although differences were noted in lamb PCV values, the reported values in the current manuscript are within range for sheep (Jackson and Cockcroft, 2002). Therefore, producers must consider the pragmatic boundaries of their respective operations to determine which management strategy should be implemented on-farm to improve the growth and health efficiencies of lambs reared in pasture-based systems.

### Lamb Growth

The current study demonstrates that lambs given a protein rich supplement while grazing on pasture known to be infected primarily with *H. contortus* had the greatest BW at the conclusion of the 112-d grazing period when compared with non-supplemented lambs grazing on pasture. This is in agreeance with previous research that illustrated that lambs consuming a basal diet of fresh forage and trickle infected with pure *H. contortus* L_3_ larvae for 10 wk had greater weight gains when supplemented with soybean meal, which provided an additional 80 g of crude protein (CP) in the diet, and compared with non-supplemented lambs (Wallace et al., 1995; Wallace et al., 1996). More recent findings from Mexico evaluating the effects of protein and energy supplementation of Pelibuey lambs artificially infected with *H. contortus* larvae reported that lambs given an additional 125 g of supplemental protein in the basal diet demonstrated greater BW gains when compared with lambs given 94 g of supplemental protein (López-Leyva et al., 2020). Lambs in the current study acquired a mixed parasitic infection naturally while grazing and were provided with a protein rich supplement that was 473 g to 490 g/kg/DM of CP. Therefore, at 1% BW, lambs in the current study were provided 135–198 g of additional CP/day. Based on the estimates provided by the small ruminant NRC (2007), 4-mo-old, late maturing, 30 kg lambs gaining 200 g/d require 125–137 g of CP/d. Under these circumstances, supplementation alone met lamb daily protein requirements and therefore excess CP received may have been further used for lamb growth. However, pasture CP was lacking, especially for new pasture in 2018. This data demonstrates that non-supplemented lambs grazing on fescue-based pastures with poor CP levels will not meet CP requirements needed to maintain growth of young, developing lambs. These experimental conditions are like those that may occur on-farm with native or unimproved pastures and thus the data suggest that providing lambs with supplemental protein in grass-based systems will further support increases in lamb growth.

Using agricultural grain byproducts as supplemental protein sources for ruminant livestock has shown to improve animal growth. In a series of experiments conducted by Felix et al. (2012), the authors demonstrated that supplementing lambs with dried distiller grains with solubles (DDGS) or soybean hulls at a rate of 2.5% BW increased lamb BW and ADG when compared with non-supplemented lambs. Moreover, supplementation feeding rate has also shown to affect lamb growth as lambs on grass pasture provided supplemental protein (190 g/kg DM) at 2% BW had greater BW gains when compared with lambs supplemented at 1% BW (Crawford et al., 2020). Although providing additional protein in the diet allows for lambs to increase BW gains on pasture, as lambs consume more supplemental feed the less forage they will consume (Newton and Young, 1974; Dove, 2002; Poli et al., 2020). When less forage is consumed, pasture utilization, forage quality, and the proportion of parasitic nematodes consumed decreases as a greater amount of residual forage and parasitic larvae remain on pasture post grazing (Dove, 2002). This notion is supported through visual observation of the grazing plots in the current study as lambs given supplemental protein had a greater amount of residual forage remaining in their paddocks at scheduled moves when compared with non-supplemented lambs. Moreover, the substitution of pasture with grain byproducts may interfere with the goals and marketing opportunities of an operation. For those marketing products labeled as grass-fed, according to the USDA (2016), livestock sold under this classification may not receive any grain or grain byproducts. Providing supplemental protein using grain derived feedstuffs would not qualify for this label, therefore producers may consider providing forages of greater nutritional quality or develop pastures with lesser parasite burdens to meet this demand.

Additionally, environmental conditions may have negatively impacted lamb growth by decreasing forage intake. It is well documented that increases in ambient air temperature and relative humidity decrease feed intake in sheep (Costa et al., 1992; Marai et al., 2007; Sevi and Caroprese, 2012). Further research that developed a panting score index for sheep illustrated that a minimum THI value of 22 resulted with visual and physiological indicators (i.e., respiration rate) of heat stress (Lees et al., 2019). As reported, a majority of the 2018 grazing season reported high THI values that were greater than 22. This data suggests that lambs in the current study were subjected to some degree of heat stress during a portion of each day, thus potentially resulting in a decrease in overall grazing activity (Osei-Amponsah et al., 2020) and lamb BW gain. Therefore, providing access to shade in the current study may have improved lamb growth. However, providing shade may have also negatively influenced lamb growth as shade reduces forage growth (Dodd et al., 2005) and may increase the instances of nematode larvae consumption due to intensive grazing near areas of shade.

In an economic assessment reviewing the financial burden of parasitic infection in ruminant based livestock production systems in Europe, Charlier et al. (2020) estimated a $2.2 billion dollar expense in agricultural production caused by gastrointestinal nematode infections with nearly 81% of the calculated losses being attributed to decreases in production efficiencies. Overall animal growth may be improved by either providing supplemental protein or removing the negative effects associated with parasitism by placing livestock on newly sown pastures that have lesser parasite burdens. Modeled after a whole farm economic analysis provided by Heard et al. (2013), our assumptions demonstrate that supplying lambs with supplemental protein while grazing on grass-based pastures resulted in improving lamb growth by 9.4 kg in 112 d. Furthermore, lambs grazing on new pasture had an increased BW of 1.3 kg when compared with lambs grazing established pasture. This figure demonstrates the benefit of decreasing the parasite burden on new pasture which resulted in a $1.02 greater net value/lamb return for those lambs grazing on newly sown pasture. Therefore, the benefit of converting cropland to pastureland within the context of the current study resulted in an increase in lamb BW, estimating the cost of parasitism at $4.69 per lamb under our assumptions provided. These assumptions are important as each producer must consider the management strategy that is best for their operation. To demonstrate comparable returns using pasture alone, forages of greater quality may be considered. However, in doing so, production costs will increase and may dilute the economic benefit of this strategy.

### Lamb Health

Lamb health as measured by variables associated with parasite resiliency may also be improved when lambs are provided supplemental protein. As previously noted, the current study demonstrates interactions between treatments when evaluating lamb FAMACHA eye score and PCV. In general, lambs given supplemental protein had lesser FAMACHA eye scores and greater PCV values when compared with non-supplemented lambs. These results are similar to those found in the current literature whereas lambs supplemented on pasture with soyhulls or DDGS had lesser FAMACHA eye scores and greater PCV values when compared with non-supplemented control lambs (Felix et al., 2012). Conversely, in evaluating the effect of different levels of protein supplementation using oaten and lucerne chaff in Boer goats administered a single dose of *H. contortus* at a rate of 100 L_3_ larvae/kg of live BW, protein supplementation did not have an effect on goat PCV (Can et al., 2017). Furthermore, regardless of infection status (infected vs. non infected), supplemental protein has shown to improve lamb PCV (Datta et al., 1998). This too is similar to the current study as lambs provided supplemental protein, regardless of pasture treatment or exposure to parasite burden, had greater final PCV values when compared with non-supplemented lambs. Focusing on the effect of parasite burden, new and established pastures were of similar quality and therefore may have had minimal impact on lamb growth and health. To improve the benefits of the newly sown pasture, the inclusion of perennial grasses, legumes, annual forages, or forage species containing condensed tannins that are of greater digestibility and quality may further aid in improving lamb growth and health efficiencies (Datta et al., 1998; Juhnke et al., 2012; Terrill et al., 2012; Campbell et al., 2021).

Protein supplementation in the current study demonstrated a reduction in overall worm burden as indicated by a decrease in lamb FEC and total abomasal nematode count. This is in support of the concept that regardless of whether the infection was experimentally induced or naturally acquired through grazing, lambs provided supplemental protein have lesser FEC when compared to non-supplemented lambs (Abbott et al., 1988; Wallace et al., 1995; Strain and Stear, 2001). Similarly, Boer does artificially infected with a single dose of 100 *H. contortus* L_3_ larvae/kg BW, demonstrated a negative correlation between FEC and supplemental protein such that FEC decreased linearly with increasing increments of supplemental protein in the basal diet, however, the *H. contortus* treatment used did not cause pathogenic effects in the Boer does (Can et al., 2017). Under conditions where sheep are continuously ingesting *H. contortus* L_3_ larvae from grazed forage, Bassetto et al. (2018) demonstrated that giving grazing ewes supplemental protein decreased ewe FEC and improved the effect of a *H. contortus* specific vaccine (Barbervax). Conversely, others have reported no differences in lamb FEC when comparing lambs fed basal diets with lambs fed basal diets supplemented with additional protein (Abbott et al., 1986; Kahn et al., 2000). Lambs provided supplemental protein in their diet demonstrated lesser nematodes observed in the abomasum at harvest when compared with lambs not supplemented with a protein rich supplement (Abbott et al., 1988; Wallace et al., 1996). It is noteworthy that the substitution effect of protein or energy supplementation on basal forage intake is rarely considered by authors in interpreting their results of protein supplementation on parasitic infections in grazed lambs. The substitution effect is the decrease in basal forage intake for a given intake of supplement (w:w) (Minson, 1990). The protein rich supplement treatment in the current study may have reduced the lamb’s intake of grazed forage. A decrease in forage intake reduces the ingestion of nematodes when compared with lambs consuming their total diet from grazed forage. The reduction in FEC, abomasal nematode numbers, and the observed lack of difference in the inflammatory response in abomasal tissue of lambs given the protein rich supplement indicates that the substitution of basal forage intake for the protein supplement may be a contributing cause of the effects associated with protein supplemented lambs in the current study.

The challenge of establishing a new pasture that is free of parasite burden was difficult to achieve. The current literature makes note of this issue as others have also attempted to create pastures free of parasitic nematodes but have failed to demonstrate a total eradication of parasites (Waller, 2006; Kidane et al., 2010; Moss et al., 2011). The new pasture in the current study was removed from crop production prior to use and had never been previously grazed by livestock according to farm records. To ensure forage establishment, the new pasture was managed mechanically for the first 2 yr. On-farm observations indicated that prior to grazing the newly sown pasture parasite status may have become compromised due to topography and deer interaction with the experimental site. Spring precipitation flooded portions of the new pasture and deposited sheep manure from adjacent pastures. Furthermore, wild populations of whitetail deer (*Odocoileus virginianus*) were commonly observed grazing in the new pasture despite our best efforts to exclude them from the area and therefore could have also further confounded our attempts in maintaining a low parasite burden pasture. However, as supported by total nematode counts and histological assessments of lamb abomasal tissue, there was a clear difference in pasture parasite burden between new and established pastures.

## SUMMARY AND CONCLUSIONS

The current study demonstrates that protein supplementation from plant-derived sources increases lamb BW and improves variables associated with parasitism evaluation. However, the exact mechanism of decreased parasite burden with additional protein supplementation remains unknown and warrants further investigation. In addition, lambs grazing pastures that have a lesser parasite burden also showed an increase in lamb BW and a decrease in total worm burden as demonstrated by lesser FEC and total nematode counts when compared with lambs grazing pasture known to have a greater parasite burden. Establishing new pastures through soil cultivation, cropland renovation, and limiting grazing access in selected pastures has shown to reduce the overall parasitic nematode burden on pasture. However, these pasture establishment practices, while not eliminating the potential for animals to acquire a parasitic infection, require a greater initial investment. Therefore, producers considering this management approach must calculate the estimated cost of each management strategy to determine which practice best fits their operation. The gross margin analysis demonstrates a greater efficiency in financial management when protein supplementation is used as a strategy to increase lamb growth and decrease parasite burden when compared with the establishment of a new pasture. Continuing to develop and implement additional management strategies will be key in reducing future use of anthelmintics. Protein supplementation and pasture maintenance are just two of many management strategies that could be utilized on-farm to create an effective parasite management program in which reduces anthelmintic use.

## References

[CIT0001] Abbott, E. M., J. J. Parkins, and P. H. Holmes. 1986. The effect of dietary protein on the pathogenesis of acute ovine heamonchosis. Vet. Parasitol. 20:275–289. doi:10.1016/0304-4017(86)90126-3 3716173

[CIT0002] Abbott, E. M., J. J. Parkins, and P. H. Holmes. 1988. Influence of dietary protein on the pathophysiology of haemonchosis in lambs given continuous infections. Res. Vet. Sci. 45:41–49. 3222552

[CIT0003] Bassetto, C. C., F. A. Almeida, G. F. J. Newlands, W. D. Smith, A. M. Castilhos, S. Fernandes, E. R. Siqueira, and A. F. T. Amarante. 2018. Trials with the *Haemonchus* vaccine, Barbervax^®^, in ewes and lambs in a tropical environment: nutrient supplementation improves protection in periparturient ewes. Vet. Parasitol. 264:52–57. doi:10.1016/j.vetpar.2018.11.006. 30503092

[CIT0004] Calvete, C., R. Calavia, L. M. Ferrer, J. J. Ramos, D. Lacasta, and J. Uriarte. 2012. Management and environmental factors related to benzimidazole resistance in sheep nematodes in Northeast Spain. Vet. Parasitol. 184:193–203. doi:10.1016/j.vetpar.2011.08.020. 21889265

[CIT0005] Campbell, B. J., C. H. Gelley, J. S. McCutcheon, F. L. Fluharty, and A. J. Parker. 2021. A comparison of annual forages and stockpiled pasture on the growth and health parameters of grazing fall-born lambs. Small Rumin. Res. 196:1–7. doi:10.1016/j.smallrumres.2021.106335

[CIT0006] Campbell, B. J., A. N. Pullin, M. D. Pairis-Garcia, J. S. McCutcheon, G. D. Lowe, M. R. Campler, and F. L. Fluharty. 2017. The effects of alternative weaning strategies on lamb health and performance. Small Rumin. Res. 156:57–65. doi:10.1016/j.smallrumres.2017.09.006

[CIT0007] Can, T. V., M. A. Hohenhaus, and P. J. Murray. 2017. The effect of different levels of crude protein on the pathophysiology of *Haemonchus contortus* infection in 2-year-old Boer dry does under confined conditions. Anim. Prod. Sci. 57:719–725. doi:10.1071/AN15024

[CIT0008] Charlier, J., L. Rinaldi, V. Musella, H. W. Ploeger, C. Chartier, H. R. Vineer, B. Hinney, G. von Samson-Himmelstjerna, B. Băcescu, M. Mickiewicz, et al. 2020. Initial assessment of the economic burden of major parasitic helminth infections to the ruminant livestock industry in Europe. Prev. Vet. Med. 182:105103. doi:10.1016/j.prevetmed.2020.105103 32750638

[CIT0009] Costa, M. J. R. P., R. G. Silva, and R. C. Souza. 1992. Effect of air temperature and humidity on ingestive behaviour of sheep. Int. J. Biometeorol. 36:218–222. 1428223 10.1007/BF02726401

[CIT0010] Crawford, C. D., D. J. Mata-Padrino, D. P. Belesky, and S. A. Bowdridge. 2020. Effects of supplementation containing rumen by-pass protein on parasitism in grazing lambs. Small Rum. Res. 190:1–9. doi:10.1016/j.smallrumres.2020.106161

[CIT0011] Datta, F. U., J. V. Nolan, J. B. Rowe, and G. D. Gray. 1998. Protein supplementation improves the performance of parasitised sheep fed a straw-based diet. Int. J. Parasitol. 28:1269–1278. doi:10.1016/s0020-7519(98)00104-0. 9762574

[CIT0012] Dodd, M. B., A. W. McGowan, I. L. Power, and B. S. Thorrold. 2005. Effects of variation in shade level, shade duration and light quality on perennial pastures. New Zeal. J. Agri. Res. 48:531–543. doi:10.1080/00288233.2005.9513686

[CIT0013] Dove, H . 2002. Principles of supplementary feeding in sheep-grazing systems. In: M. Freer, H. Dove, editors, Sheep nutrition. CABI Pub. in Association with CSIRO; p. 119–141.

[CIT0014] Falzon, L. C., P. I. Menzies, J. Vanleeuwen, A. Jones-Bitton, K. P. Shakya, J. Avula, J. T. Jansen, and A. S. Peregrine. 2013. A survey of farm management practices and their associations with anthelmintic resistance in sheep flocks in Ontario, Canada. Small Rum. Res. 114:41–45. doi:10.1016/j.smallrumres.2013.06.007 23218224

[CIT0015] FASS. 2010. Guide for the care and use of agricultural animals in agricultural research and teaching. consortium for developing a guide for the care and use of agricultural animals in agricultural research and teaching. 3rd ed. Champaign, IL: Fed. Anim. Sci. Soc.

[CIT0016] Felix, T. L., I. Susin, L. M. Shoup, A. E. Radunz, and S. C. Loerch. 2012. Effects of supplemental dried distillers grains or soybean hulls on growth and internal parasite status of grazing lambs. Sheep Goat Res. 27:1–8.

[CIT0017] Heard, J., B. Malcolm, T. Jackson, J. Tocker, P. Graham, and A. White. 2013. Whole farm analysis versus activity gross margin analysis: a sheep farm example. AFBM J. 10:16–29.

[CIT0018] Houdijk, J. G., I. Kyriazakis, A. Kidane, and S. Athanasiadou. 2012. Manipulating small ruminant parasite epidemiology through the combination of nutritional strategies. Vet. Parasitol. 186:38–50. doi:10.1016/j.vetpar.2011.11.044 22154256

[CIT0019] Jackson, P. G. G., and P. D. Crokcroft. 2002. Appendix 2 laboratory reference values: haematology. Clinical Examination of Farm Animals. Available from https://onlinelibrary.wiley.com/doi/pdf/10.1002/9780470752425.app2. (Accessed: 24 February 2021).

[CIT0020] Juhnke, J., J. Miller, J. O. Hall, F. D. Provenza, and J. J. Villalba. 2012. Preference for condensed tannins by sheep in response to challenge infection with *Haemonchus contortus*. Vet. Parasitol. 188:104–114. doi:10.1016/j.vetpar.2012.02.015 22459112

[CIT0021] Jurasek, M. E., J. K. Bishop-Stewart, B. E. Storey, R. M. Kaplan, and M. L. Kent. 2010. Modification and further evaluation of fluorescein-labeled peanut agglutinin test for identification of *Haemonchus contortus* eggs. Vet. Parasitol. 169:209–213. doi:10.1016/j.vetpar.2009.12.003 20060646

[CIT0022] Kahn, L. P., I. Kyriazakis, F. Jackson, and R. L. Coop. 2000. Temporal effects of protein nutrition on the growth and immunity of lambs infected with *Trichostrongylus colubriformis*. Int. J. Parasitol. 30:193–205. doi:10.1016/s0020-7519(99)00192-7 10704602

[CIT0023] Kaplan, R. M., and A. N. Vidyashankar. 2012. An inconvenient truth: global worming and anthelmintic resistance. Vet. Parasitol. 186:70–78. doi:10.1016/j.vetpar.2011.11.048 22154968

[CIT0024] Kidane, A., J. G. Houdijk, S. Athanasiadou, B. J. Tolkamp, and I. Kyriazakis. 2010. Effects of maternal protein nutrition and subsequent grazing on chicory (*Cichorium intybus*) on parasitism and performance of lambs. J. Anim. Sci. 88:1513–1521. doi:10.2527/jas.2009-2530 20023143

[CIT0025] Lees, A. M., M. L. Sullivan, J. C. W. Olm, A. J. Cawdell-Smith, and J. B. Gaughan. 2019. A panting score index for sheep. Int. J. Biometeorol. 63:973–978. doi:10.1007/s00484-019-01711-3 30911881

[CIT0026] López-Leyva, Y., R. González-Garduño, M. Huerta-Bravo, R. Ramírez-Valverde, G. Torres-Herández, J. Arece-García, and M. E. Lópex-Arellano. 2020. High energy levels in the diet reduce the parasitic effect of *Haemonchus contortus* in Pelibuey sheep. Heliyon. 6:1–7. doi:10.1016/j.heliyon.2020.e05870 PMC7785848 33426348

[CIT0027] Marai, I. F. M., A. A. El-Darawany, A. Fadiel, and M. A. M. Abdel-Hafez. 2007. Physiological traits as affect by heat stress in sheep – A review. Small Rumin. Res. 71:1–12. doi:10.1016/j.smallrumres.2006.10.003

[CIT0028] Michel, J. F . 1985. Strategies for the use of anthelmintics in livestock and their implications for the development of drug resistance. Parasitology 90 (Pt 4):621–628. doi:10.1017/s0031182000052276. 3892435

[CIT0029] Minson, D. J . 1990. Forage in ruminant nutrition. San Diego, California: Acad. Press Inc. 92101.

[CIT0030] Moss, R. A., R. A. Dynes, and C. L. Goulter. 2011. Effect of herbage species and renewal technique on the free living stages of gastro-intestinal roundworms. New Zeal. J. Agri. Res. 54:15–22. doi:10.1080/00288233.2010.535488

[CIT0031] Newton, J. E., and N. E. Young. 1974. The performance and intake of weaned lambs grazing S24 perennial ryegrass, with and without supplementation. Anim. Prod. 18:191–199. doi:10.1017/S0003356100017438

[CIT0032] NRC. 2007. Nutrient requirements of small ruminants: sheep, goats, cervids, and new world camelids. Washington, DC: Natl. Acad. Press.

[CIT0033] Osei-Amponsah, R., F. R. Dunshea, B. J. Leury, L. Cheng, B. Cullen, A. Joy, A. Abhijith, M. H. Zhang, and S. S. Chauhan. 2020. Heat stress impacts on lactating cows grazing Australian summer pastures on an automatic robotic dairy. Animals. 10:1–12. doi:10.3390/ani10050869 PMC7278445 32429603

[CIT0034] Palmer, D. G., and I. L. McCombe. 1996. Lectin staining of trichostrongylid nematode eggs of sheep: rapid identification of *Haemonchus contortus* eggs with peanut agglutinin. Int. J. Parasitol. 26:447–450. doi:10.1016/0020-7519(96)00009-4 8773534

[CIT0035] Poli, C. H. E. C., A. L. G. Monteiro, T. Devincenzi, F. H. M. A. R. de Albuquerque, J. H. da Motta, L. I. Borges, and J. P. Muir. 2020. Management strategies for lamb production on pasture-based systems in subtropical regions: a review. Front. Vet. Sci. 7:543. doi:10.3389/fvets.2020.00543 33102541 PMC7522395

[CIT0036] Sevi, A., and M. Caroprese. 2012. Impact of heat stress on milk production, immunity and udder health in sheep: a critical review. Small Rumin. Res. 107:1–7. doi:10.1016/j.smallrumres.2012.07.012

[CIT0037] Strain, S. A., and M. J. Stear. 2001. The influence of protein supplementation on the immune response to *Haemonchus contortus*. Parasite Immunol. 23:527–531. doi:10.1046/j.1365-3024.2001.00410.x 11696163

[CIT0038] Taylor, M. A . 2012. Emerging parasitic diseases of sheep. Vet. Parasitol. 189:2–7. doi:10.1016/j.vetpar.2012.03.027 22525586

[CIT0039] Terrill, T. H., J. E. Miller, J. M. Burke, J. A. Mosjidis, and R. M. Kaplan. 2012. Experiences with integrated concepts for the control of *Haemonchus contortus* in sheep and goats in the United States. Vet. Parasitol. 186:28–37. doi:10.1016/j.vetpar.2011.11.043 22178411

[CIT0040] Torres-Acosta, J. F., P. Mendoza-de-Gives, A. J. Aguilar-Caballero, and J. A. Cuéllar-Ordaz. 2012. Anthelmintic resistance in sheep farms: update of the situation in the American continent. Vet. Parasitol. 189:89–96. doi:10.1016/j.vetpar.2012.03.037 22520233

[CIT0041] Wallace, D. S., K. Bairden, J. L. Duncan, G. Fishwick, M. Gill, P. H. Holmes, Q. A. McKellar, M. Murray, J. J. Parkins, and M. J. Stear. 1995. Influence of supplementation with dietary soyabean meal on resistance to haemonchosis in Hampshire down lambs. Res. Vet. Sci. 58:232–237. doi:10.1016/0034-5288(95)90108-6 7659847

[CIT0042] Wallace, D. S., K. Bairden, J. L. Duncan, G. Fishwick, M. Gill, P. H. Holmes, Q. A. McKellar, M. Murray, J. J. Parkins, and M. Stear. 1996. Influence of soyabean meal supplementation on the resistance of Scottish blackface lambs to haemonchosis. Res. Vet. Sci. 60:138–143. doi:10.1016/s0034-5288(96)90008-9 8685535

[CIT0043] Waller, P. J . 1997. Anthelmintic resistance. Vet. Parasitol. 72:391–405; discussion 405. doi:10.1016/s0304-4017(97)00107-6. 9460208

[CIT0044] Waller, P. J . 2006. Sustainable nematode parasite control strategies for ruminant livestock by grazing management and biological control. Anim. Feed Sci. Technol. 126:277–289. doi:10.1016/j.anifeedsci.2005.08.007

[CIT0045] Waller, P. J., and P. Chandrawathani. 2005. *Haemonchus contortus*: parasite problem No. 1 from Tropics – Polar Circle. Problems and prospects for control based on epidemiology. Trop. Biomed. 22:131–137. 16883278

[CIT0046] Wood, I. B., N. K. Amaral, K. Bairden, J. L. Duncan, T. Kassai, J. B. Malone, Jr, J. A. Pankavich, R. K. Reinecke, O. Slocombe, and S. M. Taylor. 1995. World Association for the Advancement of Veterinary Parasitology (W.A.A.V.P.) second edition of guidelines for evaluating the efficacy of anthelmintics in ruminants (bovine, ovine, caprine). Vet. Parasitol. 58:181–213. doi:10.1016/0304-4017(95)00806-2. 7571325

